# Engineering of a plasmid-free *Escherichia coli *strain for improved *in vivo *biosynthesis of astaxanthin

**DOI:** 10.1186/1475-2859-10-29

**Published:** 2011-04-26

**Authors:** Karin Lemuth, Kristin Steuer, Christoph Albermann

**Affiliations:** 1 Current Address: Fraunhofer IGB, Nobelstrasse 12, 70569 Stuttgart, Germany; 2 Institute of Microbiology, Universität Stuttgart, Allmandring 31, 70569 Stuttgart, Germany

**Keywords:** astaxanthin, chromosomal integration, *E. coli*

## Abstract

**Background:**

The xanthophyll astaxanthin is a high-value compound with applications in the nutraceutical, cosmetic, food, and animal feed industries. Besides chemical synthesis and extraction from naturally producing organisms like *Haematococcus pluvialis*, heterologous biosynthesis in non-carotenogenic microorganisms like *Escherichia coli*, is a promising alternative for sustainable production of natural astaxanthin. Recent achievements in the metabolic engineering of *E. coli *strains have led to a significant increase in the productivity of carotenoids like lycopene or β-carotene by increasing the metabolic flux towards the isoprenoid precursors. For the heterologous biosynthesis of astaxanthin in *E. coli*, however, the conversion of β-carotene to astaxanthin is obviously the most critical step towards an efficient biosynthesis of astaxanthin.

**Results:**

Here we report the construction of the first plasmid-free *E. coli *strain that produces astaxanthin as the sole carotenoid compound with a yield of 1.4 mg/g cdw (*E. coli *BW-ASTA). This engineered *E. coli *strain harbors xanthophyll biosynthetic genes from *Pantoea ananatis *and *Nostoc punctiforme *as individual expression cassettes on the chromosome and is based on a β-carotene-producing strain (*E. coli *BW-CARO) recently developed in our lab. *E. coli *BW-CARO has an enhanced biosynthesis of the isoprenoid precursor isopentenyl diphosphate (IPP) and produces β-carotene in a concentration of 6.2 mg/g cdw. The expression of *crtEBIY *along with the β-carotene-ketolase gene *crtW148 *(NpF4798) and the β-carotene-hydroxylase gene (*crtZ*) under controlled expression conditions in *E. coli *BW-ASTA directed the pathway exclusively towards the desired product astaxanthin (1.4 mg/g cdw).

**Conclusions:**

By using the λ-Red recombineering technique, genes encoding for the astaxanthin biosynthesis pathway were stably integrated into the chromosome of *E. coli*. The expression levels of chromosomal integrated recombinant biosynthetic genes were varied and adjusted to improve the ratios of carotenoids produced by this *E. coli *strain. The strategy presented, which combines chromosomal integration of biosynthetic genes with the possibility of adjusting expression by using different promoters, might be useful as a general approach for the construction of stable heterologous production strains synthesizing natural products. This is the case especially for heterologous pathways where excessive protein overexpression is a hindrance.

## Background

Xanthophylls comprise the group of oxygenated carotenoids that are synthesized by many photosynthetic organisms and also by some non-photosynthetic yeasts, fungi, and bacteria via condensation of isoprenoid units and subsequent oxidation reactions. The xanthophyll astaxanthin (3,3'-dihydroxy-β,β-carotene-4,4'-dione) has gained considerable attention due to its beneficial effect on human health. It has been shown that astaxanthin bears a strong antioxidant and singlet oxygen-quenching activity [1] which can modulate biological functions ranging from lipid peroxidation to tissue protection against UV-light damage [2]. Preclinical studies have further shown that astaxanthin exhibits anti-inflammatory properties and reduces rethrombosis after thrombolysis [3]. Even more than the encouraging beneficial health properties, astaxanthin is used as a food colorant. The red-orange color of astaxanthin is closely connected with the quality of salmon or trout, for example. Therefore, the supplementation of astaxanthin or other carotenoids to their diets improves their value. Furthermore, the natural carotenoids in the diet of fish play an important role in reproduction [4].

**Figure 1:**
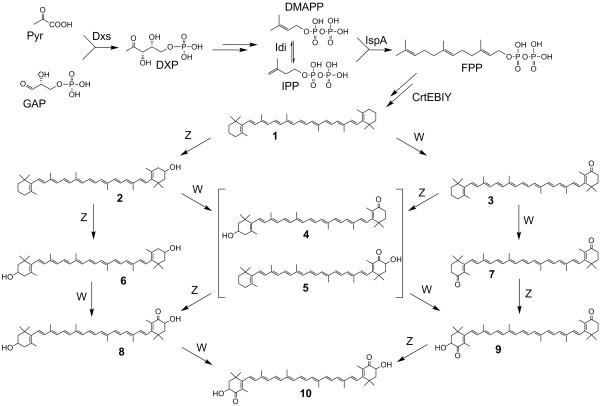
**Scheme of the putative astaxanthin biosynthesis pathway in recombinant *E. coli*, proceeding from the central intermediates pyruvate (Pyr) and glyceraldehyde-3-phosphate (GAP), highlighting the different possible routes of ketolase and hydroxylase reaction that can take place by the conversion of β-carotene (1) to astaxanthin (10)**. Dxs 1-deoxyxylulose-5-phosphate synthase, Idi isopentenyl diphosphate isomerise, IspA farnesyl diphosphate synthase, CrtE geranylgeranyl diphosphate synthase, CrtB phytoene synthase, CrtI phytoene desaturase, CrtY lycopene cyclase, W β-carotene ketolase (CrtW), Z β-carotene hydroxylase (CrtZ), IPP isopentenyl diphosphate, DMAPP dimethylallyl diphosphate, DXP 1-deoxyxylulose-5-phosphate, (**2**) β-cryptoxanthin, (**3**) echinenone, (**4**) 3'-hydroxyechinenone, (**5**) 3-hydroxyechinenone, (**6**) zeaxanthin, (**7**) canthaxanthin, (**8**) adonixanthin, (**9**) adonirubin.

Besides chemical synthesis or extraction from naturally producing organisms like the green algae *Haematococcus pluvialis *or the yeast *Xanthophyllomyces dendrorhous *[5], the use of non-carotenogenic microorganisms like *Escherichia coli *as a heterologous biosynthesis host is a promising alternative for the sustainable production of natural astaxanthin. Recent achievements in metabolic engineering of *E. coli *and yeast strains have led to a significant increase in the productivity of isoprenoid compounds (in particular terpenoids and carotenoids), by increasing the metabolic flux towards the isoprenoid precursors [6-11]. For the heterologous biosynthesis of astaxanthin, however, the complete conversion of β-carotene to astaxanthin is obviously the most critical step towards an efficient biosynthesis of astaxanthin in *E. coli *[12-14]. The conversion of β-carotene to astaxanthin requires the introduction of keto-groups at 4 and 4' as well as hydroxyl-groups at 3 and 3'positions of the β-ionone rings. The addition of the keto groups is catalyzed by the β-carotene ketolase, which is encoded by *crtO *or by *crtW *genes [14,15]. The introduction of the hydroxyl groups is catalyzed by the β-carotene hydroxylase. There are three known isoforms of this enzyme: CrtZ, CrtR, and cytochrome-P450 hydroxylase [16-19]. The isoforms of β-carotene ketolase and β-carotene hydroxylase as well as their variants from different organisms show differences in their substrate specificity concerning the 3-hydroxylation or 4-ketolation status of the β-ionone rings. As a result, the expression of ketolase and hydroxylase in naturally producing organisms as well as in a heterologous host leads to the formation of up to eight intermediates beside astaxanthin. See Figure 1 for details of astaxanthin biosynthesis in a non-carotinogenic strain like *E. coli*. *E. coli *produces farnesyl pyrophosphate (FPP) as an isoprenoid precursor that can be further converted to astaxanthin by heterologous biosynthetic genes. In the cyanobacterium *Nostoc puntiforme *PCC73102 two different genes of the *crtW*-type were found (NpF4798 (*crtW148*), NpF5919 (*crtW38*)). The recombinant enzymes CrtW38 and CrtW148 are both able to convert β-carotene into canthaxanthin, but only CrtW148 converts zeaxanthin into astaxanthin [20]. Similar observations have been made in the case of hydroxylase enzymes. CrtZ from *Pantoea ananatis *is able to introduce hydroxyl-groups into the β-ionone rings regardless of the presence of keto-groups in 4 and 4' positions [12]. The structurally different hydroxylases from cyanobacteria are not able to convert echinenone or canthaxanthin into astaxanthin [21].

At present, no recombinant xanthophyll-producing *E. coli *host has been reported that is able to accumulate astaxanthin as the sole carotenoid compound. However, significant improvements have been achieved by protein engineering of the β-carotene ketolase. By using random mutagenesis, *crtW *mutants form *Paracoccus sp*. and *Sphingomonas sp*., were generated that showed an up to 81% [22] and 90% [23] production of astaxanthin, respectively, when expressed in zeaxanthin-producing *E. coli *strains.

So far, all studies on the formation of xanthophyll compounds in *E. coli *use plasmids for the expression of the heterologous genes. To avoid the use of recombinant plasmids and to allow dispensing with selection makers like antibiotics, we present here a strain harboring the biosynthetic genes *crtE, crtB, crtI, crtY*, and *crtZ *from *P. ananatis *and *crtW148 *(NpF4798) from *N. punctiforme *PCC 73102 that are required for the formation of astaxanthin in *E. coli *stably inserted into the chromosome, along with an enhanced expression of the native *E. coli *genes *idi *and *dxs*. This strain is based on a β-carotene-producing basis strain (*E. coli *BW-CARO) recently developed in our lab [24]. Furthermore, by balancing the expression of *crtZ *and *crtW148*, which was determined by reverse transcription quantitative real-time polymerase chain reaction (RT-qPCR), a plasmid-free *E. coli *strain was engineered that accumulated astaxanthin as the exclusive carotenoid.

## Materials and methods

### Bacterial strains, media, and chemicals

All strains and plasmids used or constructed in this study are listed in table 1. The bacterial strains used in this study were *Escherichia coli *BW-CARO [24] and *E. coli *DH5α. If not stated otherwise, strains of *E. coli *were grown at 37°C in LB medium. The minimal medium used had the following composition: KH_2_PO_4 _3 g L^-1^, K_2_HPO_4 _12 g L^-1^, (NH_4_)_2_SO_4 _5 g L^-1^, MgSO_4 _× 7H_2_O 0.3 g L^-1^, CaCl_2 _× 2H_2_O 0.015 g L^-1^, NaCl 0.1 g L^-1^, Glucose monohydrate 5 g L^-1^, FeSO_4 _× 7H_2_O/sodium citrate 15 ml L^-1 ^(from the solution of 7.5 g L^-1 ^FeSO_4 _× 7H_2_O and 100 g L^-1 ^sodium citrate), thiamine, trace elements [25] 33 ml L^-1^. Antibiotics were used at the following final concentrations: ampicillin 100 μg ml^-1^, chloramphenicol 50 μg ml^-1^. Difco MacConkey agar base was purchased from Nordwald (Hamburg, Germany). Carotenoid standards were provided by the Institute of Technical Biochemistry, University of Stuttgart. All other chemicals and reagents are from Sigma-Aldrich or Roth (Germany) and were of the highest available purity.

**Table 1:** Bacterial strains and plasmids

Strains	Relevant genotype or sequences	Source
*E. coli *DH5α	F^-^, ϕ80d, *lacZ*ΔM15, *endA*1, *recA*1, *hsdR*17(r_K_^-^m_K_^-^), *supE*44, *thi*-1, *gyrA*96, *relA*1, Δ(*lacZYA-argF*)U169	laboratory strain
*E. coli *BW-CARO	*E. coli *BW25113, *malEG::crtE, fucIK::crtB*, *xylAB::crtI, lacZA::crtY*	[24]
*E. coli *BW-CARO*dxs*	*E. coli *BW25113, *malEG::crtE, fucIK::crtB*, *xylAB::crtI, lacZA::crtY*, P_T5_*-dxs*	this study
*E. coli *BW-CARO*dxs-idi*	*E. coli *BW25113, *malEG::crtE, fucIK::crtB*, *xylAB::crtI, lacZYA::crtY*, P_T5_*-dxs, galEM::idi*	this study
*E. coli *BW-CANT	*E. coli *BW25113, *malEG::crtE, fucIK::crtB*, *xylAB::crtI, lacZA::crtY*, P_T5_*-dxs, galEM::idi, melAB::crtW148 *	this study
*E. coli *BW-ASTA	*E. coli *BW25113, *malEG:: *P_tac_*-crtE, fucIK::*P_tac_*-crtB*, *xylAB::*P_tac_*-crtI, lacZA::*P_tac_*-crtY*, P_T5_*-dxs, galEM:: *P_tac_*-idi, melAB::*P_tac_*-crtW148, rbsDK::*P_rha_*-crtZ *	this study
Plasmids		
pKD46	P_araB _γ β exo (red recombinase), Amp^R^	[29]
pPQE32-148	pPQE32, *N. punctiforme *PCC 73102 *crtW148148 *gene, Amp^R^	[20]
pJF119ΔN	cloning vector, RBS, P_tac_, Amp^R^	[26]
pCAS1	pET11a, *P. putida KT2440 hpd *gene, Amp^R^,	[26]
pJF119-idi	pJF119ΔN, *E. coli idi *gene, Amp^R^	this study
pJF119-idi-FRT-*cat*-FRT	pJF119ΔN, *E. coli idi *gene, FRT-sites, Amp^R^, Cm^R^	this study
pQE31	cloning vector, RBS, P_T5_, Amp^R^	Qiagen, Germany
pCAS30-FRT-*cat*-FRT	pJF119ΔN, *P. ananatis crtE *gene, FRT-sites, Amp^R^, Cm^R^	[27]
pJF119-*crtW148*	pJF119ΔN, *N. punctiforme crtW148 *gene, Amp^R^,	this study
pJF119-*crtW148*-FRT-*cat*-FRT	pJF119ΔN, *N. punctiforme crtW148 *gene, FRT-sites, Amp^R^, Cm^R^	this study
pAW-*crtZ*	pAW223, *P. ananatis crtZ *gene, Amp^R^	this study
pAW-*crtZ*-FRT-*cat*-FRT	pAW223, *P. ananatis crtZ *gene, FRT-sites, Amp^R^, Cm^R^	this study

### Construction of plasmids

The *crtW148 *reading frame was obtained from plasmid pPQE32-148 [20]. After *Nde*I/*Hind*III digestion, the fragment was ligated into *Nde*I/*Hind*III digested pJF119ΔN [26] and transformed into *E. coli *DH5α. The resulting plasmid pJF119-crtW148 was further treated with *Hind*III and ligated with a *Hind*III digested FRT-*cat*-FRT fragment derived from plasmid pCAS30-FRT-*cat*-FRT [27].

The reading frame of *crtZ *was isolated from plasmid pJF119-crtZ [24] after *Nde*I/*Bam*HI treatment and cloned into pAW223 [*Nde*I/*Bam*HI]. The resulting plasmid pAW-crtZ was further treated with *Hind*III and ligated with a *Hind*III digested FRT-*cat*-FRT fragment. The DNA sequence of *idi *was amplified by polymerase chain reaction (PCR) from chromosomal DNA of *E. coli *LJ110 using the following oligonucleotides: a) GTGAGAACATATGCAAACGGAACACGTC b) CAAATGTCGGGATCCTTTTATTTAAGCTGGG. The PCR-product was treated with *Nde*I and *Bam*HI and was ligated into expression vector pJF119ΔN hydrolyzed with *Nde*I and *Bam*HI. The resulting plasmid pJF-idi was further treated with *Hind*III and ligated with a *Hind*III digested FRT-*cat*-FRT fragment to gain plasmid pJF119-idi- FRT-*cat*-FRT.

The *dxs *promoter replacement was conducted according to the method used by Yuan et al [28]. Thus, for the construction of pQE31-FRT-*cat*-FRT, the DNA sequence of FRT-cat-FRT was amplified by PCR from plasmid pCAS30-FRT-*cat*-FRT by using the following oligonucleotides: a) CACAGACTGAGGATCCTCGAGAGTCGACCTGCAGG and b) CTGTTTTATCAGACCGCTCGAGCGTTCTGATTTA. The PCR product was hydrolyzed with *Xho*I and ligated into the *Xho*I hydrolyzed plasmid pQE31.

### Chromosomal integration

The individual expression cassettes of *idi*, *crtW148*, and *crtZ *from plasmid pJF-idi- FRT-*cat*-FRT, pJF-crtW-FRT-*cat*-FRT, and pAW-crtZ-FRT-*cat*-FRT, respectively, were amplified by PCR. The oligonucleotide primers for the P_tac_-*crtW *expression cassette were: 1) CGGCGCATATTGCCCTGATGGACATTGACCCCACCCGCCTGGAAGAGTCGCATATTGTTCAAGGCGCACTCCCGTTCTGG and 2) AGCGCAACGATGGCTTTAAGTGTCAGATGGCTTCCTTCAGCAGACGGTTGATTGTCTGCAGGGTTATTGTCTCATGAGCG. Primers for the P_rha_-*crtZ *expression cassette were: ACCGTTCTTAATTCTGATATTTCATCGGTGATCTCCCGTCTGGGACATACCGATATGCATGCATCGATCACCACAATT and ATTCACGCTAGCCCATACACCACGACTTCCTAAAGTAATCAGTACAGTACGGATACC CAGGGTTATTGTCTCATGAGCGGATAC. Primers for the P_tac_-*idi *expression cassette were: TGCAGGCATGAAACCGCGTCTTTTTTCAGATAAAAAGCGCAATCAGTCGCTCAA GGCGCACTCCCGTTCTGG and TAACATTACTCAGCAATAAACTGATATTCCGTCAGGCTGGAATAAGGATGGCCT TCTGCTTAATTTGATGCCTG.

For the integration of the expression cassettes, the purified PCR products were each transformed into *E. coli *BW-CARO and its variant strain carrying plasmid pKD46. Expression of the λ-Red enzymes and the preparation of competent cells were carried out as described previously [29]. Competent cells were electroporated with 0.2-0.4 μg of PCR product. After electroporation, the cells were resuspended in 1 ml LB-medium and incubated at 30°C for 12 h with shaking. Subsequently, the cell suspension was spread onto MacConkey-agar plates containing chloramphenicol and 1% of the respective sugar (D-galactose, D-ribose, or melibiose), corresponding to the targeted genes responsible for sugar degradation. MacConkey agar plates were incubated over-night at 37°C. 5-10 pale colonies per transformation were picked [24] and checked regarding correct recombination by control PCR. The chloramphenicol resistance cassette was eliminated using the plasmid pCP20 as previously described [30].

### Extraction and analysis of carotenoids

*E. coli *strains carrying carotenoid biosynthetic genes were cultivated in shake flasks in 50 ml LB medium or minimal medium. Cultivations were carried out at 30°C. At an optical density (OD_600_) of 0.3 - 0.4 the cultures were induced, if required, by addition of IPTG (0.5 mM, final conc.) and/or L-rhamnose (0.2% w/v, final conc.). Samples (1 ml) were withdrawn from cultures after different time points within a period of 48 h. The cells were harvested by centrifugation, washed with cold water, and subsequently extracted by vigorous shaking with acetone (500 μl) for 15 min at 50°C. Insoluble components of the extract were removed by centrifugation (20,000 × g). HPLC analysis was performed on Dionex HPLC Instrument (Idstein, Germany), installed with a Chromeleon Software, Gina autosampler, P580 pumps, and a diode array detector. Products were analyzed by loading 50 μl of the supernatant onto a C30-reverse-phase HPLC column (250 mm × 4.6 mm, 5 μm, YMC-Europa GmbH, Dinslaken, Germany) attached with a guard column containing matrix of the same material as the column. A solvent flow rate of 1.0 ml/min was used. The solvents used were Solvent A, consisting of MTBE (methyl-*tert*-butylether)/methanol/water (19/80/1) and Solvent B, consisting of MTBE/methanol/water (90/9/1). Gradient conditions: equilibration conditions at 2% B; 0 to 22 min linear gradient from 2% B to 100% B. The spectra of the eluted carotenoids were recorded online with a Dionex UVD340 diode array detector (200 - 600 nm).

Carotenoid compounds were identified by co-chromatography using authentic standard compounds and by analysis of its UV-Vis spectra.

For the quantification of the carotenoid compounds the integrated peak areas were compared to HPLC standard curves of authentic standards.

### Detection and quantification of *crtW148 *and *crtZ*

Although isoprenoid products of CrtW148 and CrtZ were detected, neither enzyme activity tests nor SDS-PAGE were able to verify the presence of these proteins. Therefore, mRNA from *crtW148 *and *crtZ *was detected by means of absolute RT-qPCR using an internal standard.

#### (I) Generation of the internal standard

Internal *hpd *(hydroxyphenyl-pyruvate dioxygenase gene from *Pseudomonas putida*) RNA standard was generated. This was similar to a protocol published by Schuhmacher et al. [31] with the following exceptions: as template for the internal standard, pCAS1 was used [26].

#### (II) Sampling, total RNA isolation, and cDNA synthesis

*E. coli *BW-ASTA was cultivated in shaking flasks at 37°C (140 rpm) either in LB; LB plus L-rhamnose [12 mM]; LB plus L-rhamnose [12 mM] and IPTG [0.5 mM] or in minimal medium (MM); MM plus IPTG [0.5 mM]; or MM plus IPTG [0.5 mM] and L-rhamnose [12 mM]. For transcript level quantification, samples from the late exponential phase were taken. In total, two biological replicates were processed.

A cell number - OD correlation was generated by counting *E. coli *BW-ASTA using a Neubauer counting chamber. According to this correlation, 5 × 10^8 ^cells were pipetted into twice the amount RNA protect bacteria reagent (Qiagen) and processed according to manufacturer's protocol to prevent RNA degradation. Prior to RNA isolation, cell pellets were spiked with 10 μL internal *hpd *standard RNA [6.3 × 10^9 ^copies/μL]. RNA was isolated using RNeasy Mini Kit (Qiagen, Hilden, Germany). On-column DNAse digestion was performed using RNase-free DNase set from Qiagen. RNA concentration and quality were assessed photometrically (NanoDrop ND 1000) and analyzed using the Agilent Bioanalyzer 2100. In total, RNA concentrations ranged from 396 ng/μL to 710 ng/μL. Only RNA with 260/280 nm ratios of 1.8 to 2.0 and 260/230 nm ratios greater than 1.8 were used for reverse transcription. Reverse transcription (RT) of 1 μg total RNA was performed using QuantiTect Rev. Transcription Kit (Qiagen, Hilden, Germany) in a total volume of 20 μL according to manufacturer's protocol. cDNA was stored at -80°C until further use.

#### (III) Quantitative real-time PCR

Quantitative real-time PCR (qPCR) primers were designed using Roche's online *Universal ProbeLibrary (UPL) Assay Design Center *(https://www.roche-applied-science.com/sis/rtpcr/upl/index.jsp; Roche Applied Science, Mannheim, Germany)). The specificity of primer pairs was checked using Basic Local Alignment Search Tool (BLAST; http://blast.ncbi.nlm.nih.gov/); for primer sequences, hydrolysis probes and PCR efficiency using plasmid or cDNA as template see Table 2. Primers were purchased from Metabion, Martinsried, Germany (purification HPLC); hydrolysis probes were ordered from Roche Applied Science, Mannheim, Germany.

**Table 2:** Primer and hydrolysis probe sequences for qPCR, amplicon length and PCR efficiencies

Gene	Primer 1 (5' - 3')	Primer 2 (5' - 3')	Amplicon length	Hydrolysis probe	Efficiency cDNA	Efficiency plasmid
*crtW148*	ttgtgcccaaacaattagcc	ttcctcgtggtagccaaaat	73	#119	1.88	1.83
*crtZ*	cgatctttatgccgtggttt	ggccacattcctgtactgc	72	#18	1.95	1.91
*hpd*	ctatctgatcgaccgcttcg	ccttcgatgaagttgaagtcg	66	#8	1.94	2

qPCR was performed in a LightCycler^® ^480 System using LightCycler^® ^480 multiwell plates 96 (white) and the standard run protocol UPL (software: LightCycler^® ^480 SW 1.5.0 - version 1.5.0.39). An absolute quantification (method: Abs Quant/2nd Derivative Max) was performed using plasmids pCAS1 (6718 bp, harboring *hpd*), pJF119-crtZ (5905 bp, harboring *crtZ*,) and pJF119-crtW148 (6038 bp, harboring *crtW148*) as standard. A dilution series of *Hind*III hydrolyzed plasmid DNA (10^9 ^- 10^4 ^copies/μL) in triplicate was used for standard curve generation. Copy number was calculated according to Equation 1:(1)

with N: copies × μL^-1^; N_A_: copies/mol (Avogadro constant with 6.022 × 10^23 ^× mol^-1^), Conc._hP_: concentration hydrolyzed plasmid (g × μL^-1^); MW: molecular weight (g × mol^-1^) and MW_hP_: molecular weight hydrolyzed plasmid.

#### (IV) Calculation of internal standard recovery and absolute copy numbers

The recovery (r) of the internal RNA standard (*hpd*) was calculated by dividing the experimentally determined copy number (N_hpd,quant_) of the internal standard by the copy number of the internal standard (N_hpd,applied_) originally used for spiking the samples multiplied with dilution factor D (Equation 2):(2)

where D is a dilution factor caused by the process of RNA isolation, RT, and quantification by qPCR (see Equation 3).(3)

with V_RNAα _[L]: volume used for RNA isolation; V_RNAω _[L]): sample volume after RNA isolation; V_RNA->cDNA _[L]: RNA volume used for cDNA synthesis; V_cDNA->qPCR _[L]: cDNA volume used for qPCR reaction and V_cDNA _[L]: total volume of cDNA synthesis reaction. Absolute copy numbers were calculated using Equation 4:(4)

with N_mRNA, cell _being the absolute copy number per cell and N_mRNAα _being the copy number of the mRNA of interest in total volume used for RNA isolation (50 μL).

N_mRNAα _is calculated by Equation 5:(5)

with N_mRNA,quant_: copy number estimated by qPCR.

## Results

### Construction of a β-carotene-producing strain

**Figure 2:**
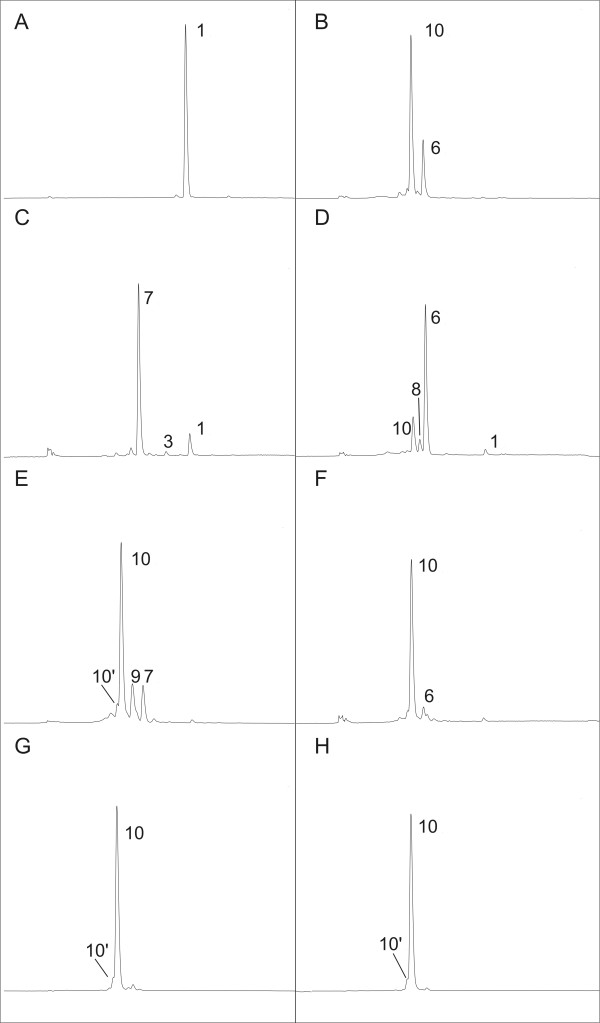
**HPLC analysis of the carotenoids accumulated during cultivation of plasmid-free *E. coli *strains**: **A **BW-CARO-dxs-idi in LB medium (48 h), **B **BW-ASTA in LB medium (48 h), **C **BW-CANT in LB medium (48 h), **D **BW-ASTA in LB medium plus L-rhamnose (48 h), **E **BW-ASTA in MM medium plus IPTG (24 h), **F **BW-ASTA in MM medium plus IPTG and L-rhamnose (48 h), **G **BW-ASTA in MM medium plus IPTG (48 h), **H **astaxanthin standard. Detection wavelength 470 nm. (**1**) β-carotene, (**3**) echinenone, (**6**) zeaxanthin, (**7**) canthaxanthin, (**8**) adonixanthin, (**9**) adonirubin, (**10**) astaxanthin, (**10'**) cis-astaxanthin.

Recently our group developed a method for the integration of heterologous expression cassettes into the chromosome of *E. coli *and the subsequent screening [24]. From this work resulted a plasmid-free strain (*E. coli *BW-CARO) carrying the four genes *crtEBIY *from *P. ananatis *in individual expression cassettes under the control of IPTG inducible *tac*-promoters. To enhance the expression level of the native 1-deoxyxylulose-5-phosphate synthase gene (*dxs*), a rate-limiting enzyme of the isoprenoid pathway [32], the native *dxs *promoter was exchanged by the strong bacteriophage promoter T5 [28]. First, a chloramphenicol resistance gene, flanked by FRT sites, was cloned upstream of the T5-promoter into pQE31. The resistance gene and promoter region of the plasmid gained (pQE31-FRT-*cat*-FRT) was PCR amplified and subsequently inserted upstream of the native *dxs *in *E. coli *BW-CARO using the λ-Red recombineering technique. After elimination of the resistance gene, the resulting strain *E. coli *BW-CARO-*dxs *was further modified by the insertion of an additional copy of the native isopentenyl diphosphate isomerase gene (*idi*), a further rate-limiting enzyme of the isoprenoid pathway [33]. The *E. coli idi *gene was cloned into the expression vector pJF119ΔN and to allow transient selection for chromosomal integration, the cloned gene was hooked up to a downstream chloramphenicol resistance cassette. The expression cassette of the resulting plasmid pJF119-FRT-*cat*-FRT, including resistance gene, was PCR amplified and transformed into *E. coli *BW-CARO-*dxs *harboring the λ-Red recombinase expression plasmid pKD46. The selected PCR primers led to the homologous recombination of the expression cassette into the galactose locus (*galETKM*). The engineered *E. coli *strains gained (*E. coli *BW-CARO, *E. coli *BW-CARO-*dxs*, and *E. coli *BW-CARO-*dxs-idi*) were cultivated in complex medium and the carotenoid content was analyzed. All strains produce β-carotene as the sole carotenoid (Figure 2A). As expected, the amount of β-carotene increased due to the enhanced expression of *dxs *and *idi*. The exchange of the native *dxs *promoter by the T5-promoter in *E. coli *BW-CARO increased the amount of β-carotene per cell by a factor of 2.6. After insertion of the additional *idi *copy into *E. coli *BW-CARO-*dxs *the amount of β-carotene per cell increased by the factor of 1.9 (β-carotene content in *E. coli *BW-CARO 1.24 ± 0.17 mg/g cellular dry weight (cdw); *E. coli *BW-CARO-*dxs *3.254 ± 0.24 mg/g cdw; *E. coli *BW-CARO-*dxs-idi *6.18 ± 0.78 mg/g cdw).

### Chromosomal integration of *crtW *and *crtZ*

The reading frame of the β-carotene ketolase gene (*crtW148*) from *N. punctiforme *PCC73102 was amplified from plasmid pQE32-148 and cloned into the expression vector pJF119ΔN, resulting in plasmid pJF-crtW148. In transformants of β-carotene-producing *E. coli *BW-CARO-*dxs-idi *with pJF-crtW148 the di-keto carotenoid canthaxanthin represented 85% (0.896 ± 0.12 mg/g cdw) of the total carotenoid content with about 10% β-carotene and small amounts of echinenone (Figure 2C). The ratio of the three carotenoids did not vary during the 48 h cultivation. The addition of the inducer IPTG had neither influence on the carotenoid formation nor on growth (data not shown).

After cloning of a FRT-*cat*-FRT cassette into pJF-crtW148, chromosomal integration of the P_tac_-crtW148 expression cassette into the melibiose locus (*melAB*) of *E. coli *BW-CARO-*dxs-idi*, and elimination of the *cat *resistance cassette, the engineered strain *E. coli *BW-CANT showed an about 20% higher formation of canthaxanthin (1.085 ± 0.15 mg/g cdw) but the percentage of canthaxanthin of all carotenoids was 85% as in the plasmid carrying strain *E. coli *BW-CARO-*dxs-idi *pJF-crtW148. The addition of IPTG up to 1 mM into LB-medium cultures of *E. coli *BW-CARO-*dxs-idi *pJF-crtW148 and *E. coli *BW-CANT, respectively, did not change the ratio of canthaxanthin, β-carotene, and echinenone as well as the total carotenoid yield, which we take as evidence of the leakiness of the *tac*-promoter in LB-medium.

In order to engineer an astaxanthin-producing strain, the β-carotene hydroxylase gene (*crtZ*) was inserted into the chromosome of *E. coli *BW-CANT. To allow a variable expression of *crtZ *compared to the *tac*-promoter controlled biosynthetic genes, *crtZ *was expressed under control of the rhamnose-promoter (P*_rhaBAD_*). For this purpose *crtZ *was cloned from plasmid pJF119-crtZ into pAW223. To verify the functional expression of this construct, pAW-crtZ was introduced into β-carotene-producing *E. coli*. Cultivation of *E. coli *BW-CARO-*dxs*-*idi *pAW-crtZ and subsequent carotenoid analysis showed that zeaxanthin was produced as the only carotenoid product (1.48 mg/g cdw).

**Figure 3:**
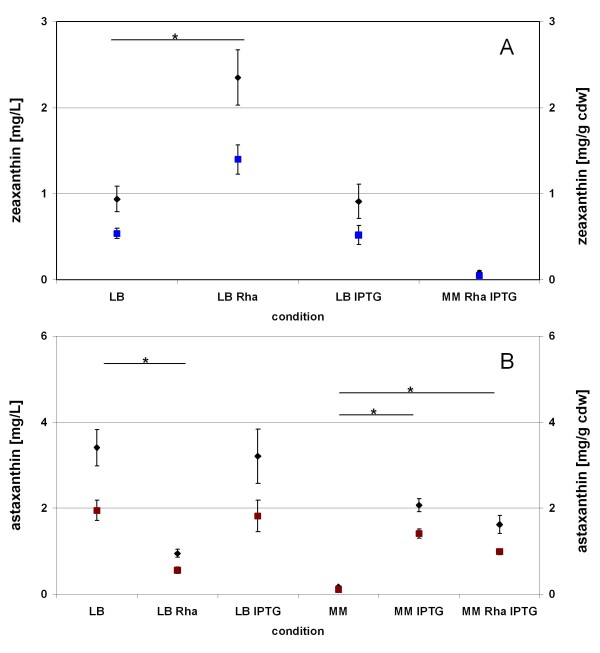
**Production of zeaxanthin (A) and astaxanthin (B) by *E. coli *BW-ASTA (in mg per liter of culture (rhomboid)) and in mg per gram cellular dry weight (cdw; (square)) after cultivation for 48 h under different conditions**. Values represent the mean (n = 4) ± standard deviation. * indicates significant changes between un-induced (LB or MM) and induced state (LB Rha, LB IPTG or MM IPTG, MM Rha IPTG, respectively). p ≤ 0.05, unpaired t-test assuming unequal variances.

Cloning of a FRT-*cat*-FRT cassette into pAW-crtZ and chromosomal integration of the P_rhaBAD_-*crtZ *expression cassette into the ribose locus (*rbsDABCK*) of *E. coli *BW-CANT and elimination of the resistance cassette resulted in the strain *E. coli *BW-ASTA, containing all required biosynthetic genes for the formation of astaxanthin as single expression units, respectively, on the chromosome. Cultivation of *E. coli *BW-ASTA in LB-medium showed that astaxanthin is the predominant carotenoid product (71%). Besides astaxanthin, zeaxanthin (25%) was the only other detectable carotenoid (Figure 2B, Figure 3). The addition of the inducer L-rhamnose to *E. coli *BW-ASTA cultures led to an increase of zeaxanthin (69%) compared to astaxanthin (20%) (Figure 2D, Figure 3). The enhanced expression of *crtZ *by L-rhamnose induction therefore led to a significant increase of zeaxanthin, obviously due to a low activity of CrtW148 for the substrate zeaxanthin. The induction of *E. coli *BW-ASTA LB medium cultures with IPTG in the presence of 0.2% L-rhamnose or without had neither effect on the amount of carotenoid nor on the ratio of astaxanthin and zeaxanthin.

### Controlled gene expression in *E. coli *BW-ASTA using glucose-containing medium

**Figure 4:**
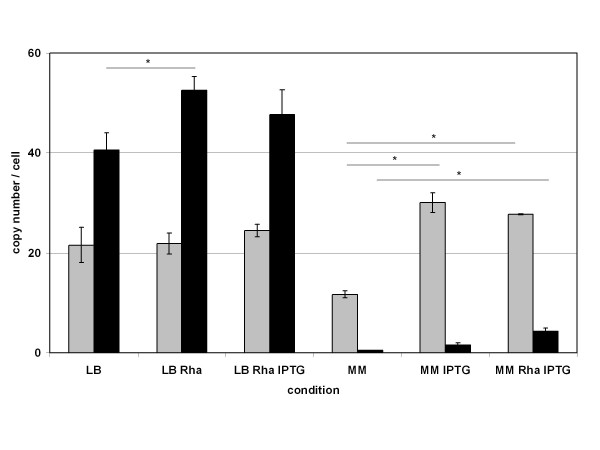
**Transcript copy numbers/cell quantified by absolute RT-qPCR**. Copy numbers ± STABW of *crtW148 *(grey) and *crtZ *(black) from *E. coli *BW-ASTA cultivated in LB or minimal medium (MM) and harvested in late exponential phase are shown. Rha: Induction with L-rhamnose [12 mM]. IPTG: Induction with IPTG [0.5 mM]. * indicates significant transcript number changes between un-induced (LB or MM) and induced state (LB Rha, LB Rha IPTG or MM IPTG, MM Rha IPTG, respectively). p ≤ 0.05, n = 4, unpaired t-test assuming unequal variances.

In order to avoid the formation of zeaxanthin by *E. coli *BW-ASTA, an increase of the ketolase activity or a decrease of the hydroxylase activity would be necessary. To better control the expression by the *tac*- and, in particular, by the *rha*-promoter that are both not tightly controlled in LB-medium (see Figure 4), minimal medium with glucose as C- and energy source was used. The cultivation of *E. coli *BW-ASTA in minimal medium without the addition of inducer molecules resulted in the formation of only small amounts (0.11 mg/g cdw) of astaxanthin, which we interpret as the result of the higher repression of the *tac*-promoter in minimal medium in contrast to LB-medium. On the other hand, the addition of IPTG to the cultures, which induces the P_tac _controlled gene expression of *dxs, idi*, *crtE*, *crtB*, *crtI*, *crtY*, and *crtW*, resulted after 48 h in a 12-fold increase in the formation of carotenoids. After 24 h of incubation in minimal medium, *E. coli *BW-ASTA synthesized 0.96 ± 0.14 mg/g cdw astaxanthin and the by-products adonirubin (13%), canthaxanthin (12%), and β-carotene (1%) (Figure 2E). In contrast to the cultivation in LB medium no zeaxanthin formation was observed. After an incubation time of 48 h astaxanthin was produced as exclusive carotenoid (>95%) in a concentration of (2.07 ± 0.15 mg/l; 1.41 ± 0.11 mg/g cdw) (Figure 3). This result shows that during the cultivation of *E. coli *BW-ASTA in minimal medium with glucose and IPTG the β-carotene produced is predominantly converted by the ketolase (CrtW148) into cantaxanthin, which is subsequently slowly hydroxylated by CrtZ. The concurrent addition of both inducers, IPTG and L-rhamnose, also led after 24 h to the formation of astaxanthin (68%), adonirubin (14%), cantaxanthin (12%), and traces of β-carotene (1%). In the late stationary phase (48 h) of the culture astaxanthin was still the predominant carotenoid with >90%, however, the cells also contained about 5% of zeaxanthin as a by-product (Figure 2F).

### Transcriptional analysis of *crtW148 *and *crtZ*

In order to ascertain whether the assumed expression levels of CrtW148 and CrtZ based on carotenoid formation by *E. coli *BW-ASTA were in accordance with the mRNA levels of *crtW148 *and *crtZ*, we performed absolute RT-qPCR studies. A method previously described by Schuhmacher et al. [31] that uses an external standard for absolute transcript number quantification, was adapted to the Roche UPL system allowing for absolute transcript number determination per cell.

From the cultivation of *E. coli *BW-ASTA in LB medium and in minimal medium, with or without induction of the *tac*- and *rha*-promoter, respectively, cells were withdrawn in the late exponential phase and were analyzed for the *crtZ *and *crtW148 *transcript level by RT-qPCR. The number of transcripts of *crtW148 *and *crtZ *under the different conditions are shown in Figure 4. In LB medium, comparable transcript numbers per cell could be detected for *crtW148 *with or without IPTG induction (between 22 ± 4 and 25 ± 1 copies/cell). A 15% higher copy number was detected for *crtZ *(41 ± 3 copies/cell compared to 48 ± 5 to 53 ± 3 copies/cell) after induction with L-rhamnose. The transcript level of *crtZ *reflects the formation of zeaxanthin when cultivated in complex medium. An increased expression of *crtZ *correlated with an increased formation of zeaxanthin by *E. coli *BW-ASTA. Under all cultivation conditions in LB medium, zeaxanthin and astaxanthin had been detected. By using glucose-containing minimal medium the transcription of the heterologous genes was more tightly regulated than in LB medium. Transcript numbers per cell of *crtW148 *without the addition of IPTG were 12 ± 1. For *crtZ *in average, less than one copy number per cell was detected in minimal medium without induction. This reflects the tight regulation of the rhamnose-promoter under these conditions. These results are in good accordance with the product formation; in minimal medium without induction, only small amounts of astaxanthin as the sole carotenoid were detected (Figure 3). The induction by IPTG led to a 2.3 to 2.6 fold increase in mRNA level of *crtW148*, and resulted in a 20-fold increase of the carotenoid concentration. Here, astaxanthin was the only carotenoid that was accumulated by *E. coli *BW-ASTA after 48 h (Figure 2). This shows that the low mRNA level of *crtZ *yielded in a sufficient amount of hydroxylase activity converting all the produced precursors into astaxanthin. The addition of L-rhamnose led to a significant increase in transcript levels of *crtZ *(8.6 fold). In these cases, both astaxanthin (90%) and zeaxanthin (5%) had been detected as products after 48 h.

## Discussion

The *in vivo *biosynthesis of a complex natural product in a heterologous host like *E. coli *first requires the introduction and the functional expression of biosynthetic genes that enable the conversion of available cell intermediates towards the desired product. The heterologous biosynthesis of astaxanthin by *E. coli *has been achieved in numerous studies by the expression of respective carotenoid biosynthetic genes using recombinant plasmids [20,23,33-36]. In this study, we constructed a plasmid-free *E. coli *strain that carries each of the astaxanthin biosynthetic genes (*crtE,B,I,Y,Z,W*) as individual expression units on the chromosome. For the integration of the expression cassettes we used a method, recently developed in our lab, that allows fast and reliable integration and screening [24]. It is based on the λ-Red mediated recombination technique developed for the directed knock-out [29]. This method utilizes the replacement of *E. coli*s' rare sugar degradation genes which are dispensable for most biotechnological applications. The replacement of these genes can easily be visualized by the use of MacConkey differential agar medium carrying the corresponding sugar compound.

The chromosomal insertion of heterologous biosynthetic genes has for obvious reasons some advantages compared to the use of heterologous plasmids. Thus, plasmids may be the best choice for the cloning and short-term expression of recombinant genes, in particular for the maximum overproduction of a given protein. Especially in metabolic engineering applications, however, a too strong gene expression may be unfavorable for long-term productivity [37]. Yoon et al. [38] observed that a high expression of lycopene biosynthetic genes in *E. coli *leads to a decrease in growth and lycopene production. Therefore, the chromosomal integration of heterologous expression cassettes can be favorably compared to multi-copy plasmids in terms of metabolic burden effects, structural instability, and most important segregational instability [39]. Furthermore, a stable chromosomal insertion obviates the use of selection-markers (e.g. antibiotics) that are commonly used for the maintenance of plasmids during cultivation. Especially antibiotics are both costly and can hamper the product purification in food and pharmaceutical production processes. On the other hand, a low enzyme activity of a heterologous downstream pathway can result in a reduced product yield or in an accumulation of pathway intermediates [40]. Thus, the *in vivo *biosynthesis of carotenoids in *E. coli *requires an appropriate heterologous gene expression, adapted to the supply of isoprenoid precursors to avoid the effect of metabolic burden and to avoid the accumulation of pathway intermediates. The increased biosynthesis of β-carotene (up to 6.2 mg/g cdw) by the enhanced expression of *idi *and *dxs *in *E. coli *BW-CARO demonstrates that the expression of the heterologous biosynthetic genes and accordingly the enzyme activity of the corresponding proteins in this strain do not limit the formation of β-carotene. Similar observations were made by Chiang et al. [41] for a lycopene-producing strain that carries a single copy of a lycopene biosynthetic gene cluster on the chromosome. Surprisingly, the additional expression of *crtW148 *and *crtZ *in the β-carotene-producing strain reduced the overall formation of carotenoids about three times compared to *E. coli *BW-CARO-*dxs*-*idi*. This leads to the suggestion that the recombinant proteins (CrtW148 or CrtZ) or a product of their enzymatic reaction effect the formation of the carotenoid precursors upstream of phytoene, because no other carotenoid accumulated in the cell. This stands in contrast to the study by Scaife et al. [36] who found that expression of a β-carotene-ketolase and -hydroxylase within a β-carotene-producing strain significantly increases the total carotenoid yield. The maximum astaxanthin yield of almost 2 mg/g in this study and ours is in the same range and represents the highest astaxanthin content in *E. coli *that has been achieved so far.

Besides the chromosomal integration, another aim of our work was to find conditions by which *E. coli *synthesizes astaxanthin as the sole carotenoid. For the conversion of β-carotene to astaxanthin, the β-carotene ketolase gene from *Nostoc punctiforme *(*crtW148*) and the β-carotene hydroxylase gene from *Pantoea ananatis *(*crtZ*) were chosen, because the corresponding proteins (CrtW148, CrtZ; see Figure 1) are known to be bifunctional and therefore accept β-carotene as well as hydroxylated or ketolated products, respectively, as substrate [12,20]. In order to vary the expression level of *crtW148 *and *crtZ*, the hydroxylase gene was expressed under control of the *rha*-promoter in contrast to the other heterologous genes that were controlled by a *tac*-promoter, respectively. The cultivation of *E. coli *BW-ASTA in LB medium showed the formation of astaxanthin as the predominant product but also a significant amount of zeaxanthin that increased upon the additional induction of the *rha*-promoter. In contrast, the additional IPTG induction of the *crtW148 *controlling *tac*-promoter had no significant influence on the product formation. The carotenoid formation is in concordance with the results of mRNA quantification. The qPCR measurement showed that both, *tac*-promoter (*crtW148*) and *rha*-promoter (*crtZ*), are only marginally repressed under the given conditions (Figure 4).

It is supposed that hydroxylase and ketolase compete for their substrate and that only a balanced expression of these two enzymes might lead to a complete conversion of β-carotene to astaxanthin [12,42-44]. This hypothesis is supported by our results. We suppose that in the astaxanthin biosynthesis by *E. coli *BW-ASTA the hydroxylation reaction occurs fast with β-carotene as well as with the ketolated intermediates as substrates. No intermediates were detected under these conditions which are mostly ketolated (Figure 2). The CrtW148 ketolase does utilize β-carotene and to a minor extent hydroxylated intermediates. But during the course of cultivation the ketolase was not able to convert the accumulated zeaxanthin into adonixanthin or astaxanthin, completely. Although the bifunctionality of CrtW148 from *N. punctiforme *was proven [20], the conversion of zeaxanthin to astaxanthin by CrtW148 is obviously the most limiting step towards the efficient biosynthesis of astaxanthin in our system. To improve the conversion of zeaxanthin to astaxanthin, protein engineering of a CrtW-type ketolase had been used successfully, but a complete transformation to astaxanthin was not achieved [22,23]. In contrast to this research, our working hypothesis was to avoid the accumulation of zeaxanthin by increasing the activity ratio of CrtW148 to CrtZ not by enhanced *crtW148 *expression or enzyme activity/specificity but instead by lowering the *crtZ *expression level. This was achieved by the use of D-glucose containing minimal medium that led to a better balanced regulation of both heterologous promoters. Especially the rhamnose-promoter, that regulates the expression of *crtZ*, is more tightly controlled by the catabolite repression [45,46]. Cultivation of *E. coli *BW-ASTA under this condition with induction of the IPTG-controlled heterologous promoters led in the early phase of the cultivation to the formation of astaxanthin and the intermediates adonirubin and canthaxanthin (Figure 2) that vanished, presumably due to the transformation into astaxanthin during the course of cultivation. We take this as evidence that the adjustment of the expression level can direct the pathway towards the desired product, astaxanthin. The low expression of *crtZ *and the, in contrast, high expression of *crtW148 *(Figure 4) make it obvious that the synthesized β-carotene is due to the kinetics of the reactions, preferentially converted into canthaxanthin, which is secondly completely transformed by the slower hydroxylation reaction via adonirubin into astaxanthin. Similar observations concerning the competition between hydroxylase and ketolase have been made for transgenic maize where plants harboring only an endogenous hydroxylase but an exogenous ketolase gene under an endospecific promoter accumulated astaxanthin. Astaxanthin, however, was not accumulated by plants harboring both exogenous hydroxylase and ketolase genes [44]. The authors indicated that the avoidance of the adonixanthin accumulation is crucial for astaxanthin production in transgenic maize endosperm [44]. In contrast, we find the avoidance of zeaxanthin accumulation to be the crucial step for sole astaxanthin synthesis in the bacterium *E. coli *BW-ASTA.

## Conclusions

In this study, we engineered a plasmid-free *E. coli *strain that carries biosynthetic genes for the *in vivo *biosynthesis of astaxanthin. The stable chromosomal insertion of the heterologous genes enables dispensing with selection makers that are required for the maintenance of recombinant plasmid. The biosynthetic genes were each integrated as single expression units into the chromosome of *E. coli*. This approach allows the control of individual gene expression levels, which is complicated to achieve if the astaxanthin biosynthetic genes are organized within one operon. This *E. coli *engineering strategy might also be useful as a general approach for the construction of stable production strains for the heterologous biosynthesis of natural products for which excessive protein overexpression is a hindrance.

The adjustment of the *crtZ *expression level in the astaxanthin-producing strain was applied successfully for the complete *in vivo *conversion of β-carotene into astaxanthin by recombinant *E. coli *cells.

## Competing interests

The authors declare that they have no competing interests.

## Authors' contributions

CA initiated and coordinated the project. KL and KS performed the experiments. KL was responsible for RT-qPCR. KL and CA wrote the paper. All authors approved the final version of the manuscript.
